# Some Human Lessons in War-Time

**Published:** 1916-10-21

**Authors:** 


### LIFE AND DEATH.

### Some Human Lessons in War-Time.

### V. THE PLEASURES OF LIFE.*

The pleasures of life are not all physical. If they were, they would be monopolised by youth and vice in man and woman, and would reduce the human being to the level of the brute beasts, whose enjoyment and pleasure are confined to the gratification of their physical instincts. One of the best definitions is Hamilton's, that "pleasure is the reflex of unimpeded energy." This upholds Martial's contention that "life is not to live, but to be well." No one can deny that one of the greatest of gifts to the human being is the continuous possession of good health. But the experienced physician knows that for a human being to be physically well is no evidence or guarantee whatever that he or she lives, in the highest meaning of the word "life." There is nothing more true than that the human being who attempts to make perpetual holiday soon experiences satiety, and a continuance in holiday-making may deprive him or her of the pleasure of life, and may ultimately cause the loss of the faculties of pleasure. Yet pleasure came from heaven, not as Young points out, to turn human brutal, but to build divine on human. The test of pleasure and its enduring value is: Does this or that pleasure give aid to reason? Will it last by fulfilling the test that it succours virtue? Once divorce pleasure from sin and excess, and it becomes an invaluable asset in the development and output of the spiritual and physical natures of men and women. The wise guard themselves against satiety by taking their enjoyment through pleasure so as not to injure those that are to follow. The fate of the perpetual pleasure-seeker is to become a slave to his senses.

### The Perfection of Pleasure.

On the other hand, a taste of pleasure undoubtedly quickens the relish of life, and the human being who can at all times sacrifice pleasure to duty, as one writer puts it, approaches sublimity. The French view—and it is sound and good—is that, the most delicate and the most sensible of all pleasures consist in promoting the pleasures of one's fellows. To live, man and woman must have a standard, and that should be the best. The highest standard is good enough, but nothing short of it is. So the perfection of pleasure is to be found initially in its wise selection, in the cultivation of the simple life, in the recognition of one's spirituality, and the acceptance of times of pleasure as the harvest time of human progress, for no real enjoyment can ever be so transient as to be merely for the moment. Every live man or woman who seizes the opportunity to travel, to study other nations, to examine the workaday life and the difficulty of existence which many of their neighbours and members of numerous classes of the population have to face; who takes the opportunity to associate with pleasant people and to reap the legitimate harvest which genuine enjoyment offers, provides his memory with a storehouse of good things, and need never be dull, for such pleasures are not transitory, but abiding.

### The Abuse of Appetite.

Without a standard no human being can really live, and very few men or women can be really well in body, mind, or spirit. Thus it is good to eat to live, but not to live to eat. How many parents, teachers, and others who have much to do with the young ever enforce this sound doctrine on their pupils? The greater the prosperity of a given nation, and the greater the prosperity of the whole world at a given time, the larger will be the number of men and women who avail themselves of the power money confers on them to gratify and abuse their appetites by beginning, continuing, and even dying of the habit of their continuous abuse. Take a large out-patient hall, full of patients—take the consulting-room of a popular practitioner—take the individual patient in a middle or upper class home—and how many of the ailments which the doctor has to treat would be absent if the overwhelming majority of the people would educate themselves not to abuse their appetites! From adolescence to old age, for example, how few ills of the flesh would remain if living to eat could be rendered impossible! What spirituality, in a real, living sense, can men or women have who habitually lay themselves out to pursue this or other degrading and destructive habits?

It is of course regrettable, however natural, that the possessors of youth, with its splendid buoyancy and magnificent and ever-increasing energy, should sometimes injure themselves in the pleasures of physical exercises. Such pleasures, however, are legitimate enough, and have the merit of being natural in young people. But there is nothing natural, or innocent, or anything but degrading, for instance, in the habit of over-feeding. It follows that, however great the transitory pleasure derived from this falling from grace may be to the individual, it must eventually, unless arrested in time, end in the physical and spiritual death of the human being who considers its indulgence a pleasure. The numerous self-deceived and piteous examples of the race who persist in living to eat never can really enjoy the pleasures of life, for there is no such thing as life derivable from such a practice, because its derivative is death. Eating may give

* Previous articles appeared on September 23 and 30, and October 7 and 14.

pleasure to the senses, but it can never bring pleasure or sustenance to the mind. Physical pleasure is sensual; mental pleasure is uplifting, recuperating, desirable, necessary. Such pleasure abides with the happy recipient of it, and, through memory and association, will bring sustenance, recuperation, and hope, whenever opportunity comes throughout the lifetime of those who are conscious of their spirituality.

The great essential to recognise is that the spirit of man must be cultivated and sustained, his mind must be fed as well as his body, or life in the highest meaning of the term is impossible, and life's span in the human being degenerates to mere existence. Human existence when dependent on the minimum of education and knowledge gravitates to mere animal existence, the fruits of which, like weeds in a neglected garden, ultimately gain the mastery, and first the individual and then the nation loses vitality, death triumphs, and the people and nation disappear. The recognition by the individual and the nation of these demonstrable truths makes for strength. The driving force of everything which makes the human being at his or her best results. They who first feed the mind set themselves to gain knowledge, to maintain knowledge, and so man, woman, and a whole people, may ultimately attain to the maximum of health and blessedness. Why should not the people of every one of the Allied nations be thus equipped at the termination of this war, each individual having become spiritually alive and awake? More and more of them are growing alive to their spirituality undoubtedly, as one great result of the war. A people so equipped cannot fail to advance. Such an awakening naturally comes to individuals alert enough to recognise the truths just stated and to set themselves to attain the maxima indicated.

Every man or woman so equipped should really enjoy life. The pleasure of life to them is real, unceasing, quite apart from their physical condition at the moment, because it rests upon the recognition and cultivation of their spiritual and physical being. Such people are like birds in the sunshine, full of song, full of life, full of the responsibility which it should bring to the human being, conscious that he or she is a trustee, for his or her life, to the Creator to Whom each will one day have to render an account.

We make bold to say that it is an absolute fact that the most alive of human beings, and the best, have the sense of trusteeship. It is to them a possession of inestimable value, because it keeps them in a state of blessedness from the circumstance that they have come to realise that the present stage of existence is a dying life, and that when death comes they will be called upon to render their account to their Creator, *i.e.*, to God Who has kept them alive through their dying life on earth. Further, of His great goodness, if they have been faithful to their trust, He will raise them up to immortality through Christ Who is the Life.

The Allied nations now are fighting for the freedom of the people of the world. They are fighting for the principles and the maintenance of the teachings of Christ as the one standard which gives to all, from the humblest to the highest, the maximum of justice, of fair dealing *inter se*, of government under which every human being whc proves faithful to his trusteeship will secure his daily bread. The writer believes and hopes that the millennium, so far as this world is concerned, may then be measurably nearer, for the majority of the people of every nation should possess spiritual and bodily health, the majority of men and women of each of the nations should rejoice in being trustees for their own lives, with the blessedness which assured hope of immortality has and does invariably bring to man and woman.

These conclusions are the result of the teachings of a long, active, and busy life which has yielded experiences of most phases of existence and of real life. They are further enforced by the incontrovertible fact that a multitude of those who have ceased to reside on earth have possessed the same vision, have been upheld through their earthly experiences, and have passed beyond the veil, not only without fear but with the most glorious anticipations, through their spirituality, of the immortality to which they have gone.

The late Bishop of Massachusetts, Dr. Phillips Brooks, in the course of a sermon on the Vision of St. John, recorded in Revelation xx. 12, "And I saw the dead, small and great, stand before God," proceeds: "Blessed are the dead who die in the Lord, and stand immortal before Him. Is the fulfilment of the vision of St. John for any man to wait until that man is dead? Can only the dead stand before God? Think for a moment what we found to be the blessings of that standing before God, and then consider that those privileges, however they may be capable of being given more richly to the soul of man in the eternal world, are privileges upon whose enjoyment any man's soul may enter here. Consider this, and the question at once is answered. Already, now, you and I may live by the standards of the eternal righteousness, and we may claim our brotherhood with the least and the greatest of our fellow-men, and we may so lay hold on God that we shall realise our immortality. The soul that has done all that is now standing before God. It does not need to push aside the curtain, and to enter into the unknown world which lies behind. While the man is living here, walking these common streets, living in closest intercourse with other men, he is already in the Everlasting Presence, and his Heaven has begun. . . . When the change comes to any of us, how little it will be, if we have really been, through the power of Jesus Christ, standing before God, in our poor, half-blind way upon the earth. If now, in the bright freshness of your youth, you give yourself to Christ, and through Him do indeed know God as your dearest Friend, years and years hence, when the curtain is drawn back for you, and you are bidden to join the host of the dead who stand before God eternally, how slight the change will be. Only the change from the struggle to the victory, only the opening of the dusk and twilight into the perfect day."

